# Urokinase System in Pathogenesis of Pulmonary Fibrosis: A Hidden Threat of COVID-19

**DOI:** 10.3390/ijms24021382

**Published:** 2023-01-10

**Authors:** Anna A. Shmakova, Vladimir S. Popov, Iliya P. Romanov, Nikita R. Khabibullin, Nailya R. Sabitova, Anna A. Karpukhina, Yana A. Kozhevnikova, Ella V. Kurilina, Zoya I. Tsokolaeva, Polina S. Klimovich, Kseniya A. Rubina, Yegor S. Vassetzky, Ekaterina V. Semina

**Affiliations:** 1 Institute of Experimental Cardiology, National Medical Research Centre of Cardiology Named after Academician E.I. Chazov, 121552 Moscow, Russia; 2 Faculty of Medicine, Lomonosov Moscow State University, 119192 Moscow, Russia; 3 Koltzov Institute of Developmental Biology, 117334 Moscow, Russia

**Keywords:** COVID-19, pulmonary fibrosis, urokinase uPA, urokinase receptor uPAR, plasminogen, scRNA-seq

## Abstract

Pulmonary fibrosis is a common and threatening post-COVID-19 complication with poorly resolved molecular mechanisms and no established treatment. The plasminogen activator system, including urokinase (uPA) and urokinase receptor (uPAR), is involved in the pathogenesis of COVID-19 and contributes to the development of lung injury and post-COVID-19 pulmonary fibrosis, although their cellular and molecular underpinnings still remain obscure. The aim of the current study was to assess the role of uPA and uPAR in the pathogenesis of pulmonary fibrosis. We analyzed uPA and uPAR expression in human lung tissues from COVID-19 patients with pulmonary fibrosis using single-cell RNA-seq and immunohistochemistry. We modeled lung fibrosis in *Plau*-/- and *Plaur*-/- mice upon bleomycin instillation and explored the effect of uPAR downregulation in A549 and BEAS-2B lung epithelial cells. We found that uPAR expression drastically decreased in the epithelial airway basal cells and monocyte/macrophage cells, whereas uPA accumulation significantly increased in tissue samples of COVID-19 patients. Lung injury and fibrosis in *Plaur*-/- vs. WT mice upon bleomycin instillation revealed that uPAR deficiency resulted in pro-fibrogenic uPA accumulation, IL-6 and ACE2 upregulation in lung tissues and was associated with severe fibrosis, weight loss and poor survival. uPAR downregulation in A549 and BEAS-2B was linked to an increased N-cadherin expression, indicating the onset of epithelial–mesenchymal transition and potentially contributing to pulmonary fibrosis. Here for the first time, we demonstrate that plasminogen treatment reversed lung fibrosis in *Plaur*-/- mice: the intravenous injection of 1 mg of plasminogen on the 21st day of bleomycin-induced fibrosis resulted in a more than a two-fold decrease in the area of lung fibrosis as compared to non-treated mice as evaluated by the 42nd day. The expression and function of the plasminogen activator system are dysregulated upon COVID-19 infection, leading to excessive pulmonary fibrosis and worsening the prognosis. The potential of plasminogen as a life-saving treatment for non-resolving post-COVID-19 pulmonary fibrosis warrants further investigation.

## 1. Introduction

December 2019 was marked by an outbreak of a new strain of coronavirus (SARS-CoV-2) in Wuhan, China, which triggered a pandemic of acute respiratory disease coronavirus 2019 (COVID-19) across the globe [1]. The unprecedented spread of COVID-19 posed a severe healthcare challenge, with long-term sequelae of severe COVID-19 cases and deaths to be encountered even after two years of the outbreak [2]. One of the most common and severe post-COVID-19 complications is pulmonary fibrosis: early evidence suggested that more than a third of COVID-19 patients discharged from hospital after recovery developed fibrotic lung abnormalities, and this percentage increased with disease severity and duration [3,4,5,6]. A reliable incidence of COVID-19-induced pulmonary fibrosis, however, can only be assessed after decades of post-COVID-19 observational studies [7,8]. Epithelial and endothelial cell damage, excessive fibrin deposition, unregulated release of cytokines and proteases, and abnormal immune system activation [9,10,11,12] ultimately result in pulmonary fibrosis with limited treatment options.

The mechanism of SARS-CoV-2 fusion with the host cell membrane requires proteolytic cleavage of the viral spike protein (S-protein) by serine proteases: primarily the transmembrane serine protease TMPRSS2 and potentially plasmin [13,14,15]. In the case of the transmembrane protease TMPRSS2, effective infection with SARS-CoV-2 requires angiotensin-converting enzyme 2 (ACE2) and TMPRSS2 to be co-expressed on the same cell membrane. While in the case of the fibrinolytic plasminogen activator system, there is still no clear picture regarding its mechanistic underpinnings in COVID-19-related pathologies. 

It is well established that the plasminogen activator system plays a crucial role in the pathogenesis of lung injury and pulmonary fibrosis. The system comprises tissue plasminogen activator (tPA), urokinase (uPA), its receptor uPAR and plasminogen activator inhibitors (PAI-1 and PAI-2) [16,17,18]. Lung fibrinolysis is driven by uPA as opposed to intravascular fibrinolysis controlled by tPA [16,18]. uPA (encoded by the *PLAU* gene) is a serine protease that cleaves inactive plasminogen, converting it into a protease plasmin with a wide range of substrate specificity (fibrin, ECM components, matrix metalloproteinases) [19]. uPA interaction with uPAR (encoded by the *PLAUR* gene) on the cell surface accelerates its catalytic activation and can trigger intracellular signal transduction [19,20,21,22]. uPAR-bound uPA cleaves plasminogen ~ six times more efficiently [23]. uPA can also be reciprocally activated by its ligand plasmin [24,25,26]. The absence of uPAR or plasminogen significantly impairs uPA activation [24]. Plasmin activation by uPA is regulated by its endogenous inhibitor, PAI-1 [18]. Lung epithelial cells express uPA, uPAR and PAI-1; alveolar macrophages and lung fibroblasts can also serve as a source of uPA in lung tissue [16]. The plasminogen activator system has been widely studied in relation to acute lung injury (for a review, see [16]); however, its role in COVID-19-induced pulmonary fibrosis remains underexplored. 

Previously, it was reported that the reduced serum levels of plasminogen and PAI-1 were significantly associated with low survival in severely ill COVID-19 patients [27,28], while high soluble uPAR serum level was shown to be an early predictor of clinical severity and poor outcome in patients with COVID-19 [29,30,31,32,33,34]. These data indicate that the plasminogen activator system is demonstrably involved in COVID-19 pathogenesis. Further research is warranted to understand the function of the plasminogen activator system in post-COVID-19 lung fibrosis.

In the current study, we aimed to define the role of uPA and uPAR in COVID-19-induced lung fibrosis with putative mechanisms of their action. We found that uPAR expression was dramatically reduced in epithelial and monocyte/macrophage cells from COVID-19 patients, while uPA extensively accumulated in the lung tissue of COVID-19 patients. Modeling the lung injury and fibrosis via bleomycin instillation in mice revealed that uPAR deficiency led to uPA excessive accumulation and an increase in IL-6 and ACE2 expression in lung tissue and was associated with severe fibrosis, weight loss and decreased survival. Moreover, uPAR downregulation in lung epithelial cells was linked to epithelial-to-mesenchymal transition (EMT). Finally, we found that mouse plasminogen intravenous administration (1 mg) could reverse lung fibrosis by reducing more than two-fold the fibrotic lung area in mice lacking uPAR expression (*Plaur*-/-) at day 42 after fibrosis induction. These findings highlight the importance of the plasminogen activator system in lung fibrosis. We proposed that plasminogen administration in COVID-19 disease may have therapeutic potential to ameliorate the persistence of fibrin and thrombosis in severely ill patients.

## 2. Results

### 2.1. uPA and uPAR Were Differentially Expressed in Lung Tissue of COVID-19 Patients

**Figure 1:**
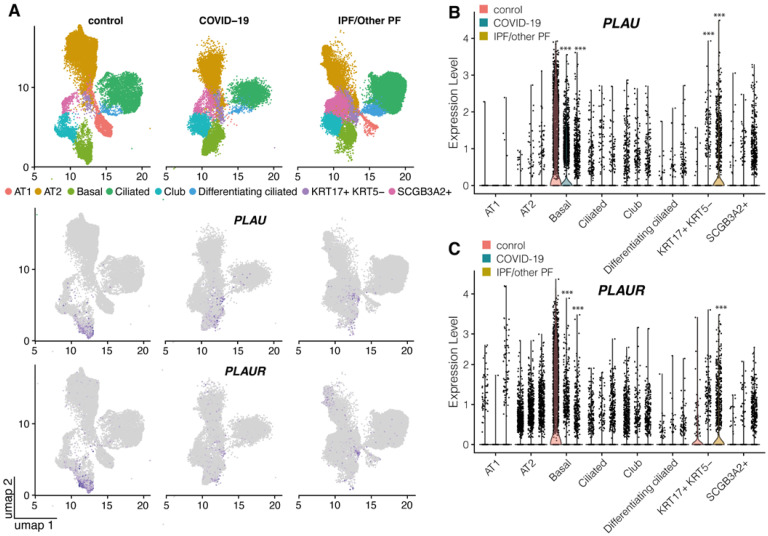
*PLAU* and *PLAUR* expression in epithelial cells of control, COVID-19 and pulmonary fibrosis (PF) lung samples: (**A**) Uniform Manifold Approximation and Projection (UMAP) embedding of jointly analyzed single-cell transcriptomes annotated by cell type (color) and disease status. Lung tissue transcriptomes of patients with COVID-19-associated pneumonia, PF and control lung samples from healthy donors, reported in Habermann et al. and Bharat et al. (GSE158127, GSE135893 [35,36]) were analyzed. Normalized expression levels of *PLAU* (**B**) and *PLAUR* (**C**) grouped by different types of epithelial cells and disease status (control, COVID-19 and PF from left to right); statistics were calculated only for cell types with the median normalized expression value > 0 with the exception of KRT17+ KRT5- cells, where the median expression value equaled to 0, but a subpopulation of *PLAU*- and *PLAUR*-positive cells still existed. *** *p_adjusted_* < 0.0001 as assessed by false discovery rate (FDR). AT1 and AT2, alveolar type I and type II cells; IPF/other PF, idiopathic pulmonary fibrosis and other pulmonary fibrosis; umap, Uniform Manifold Approximation and Projection.

We first addressed the role of uPA and uPAR in COVID-19-induced lung fibrosis through data-mining of scRNA-seq datasets from (i) warm postmortem lung biopsies of patients diseased from COVID-19-associated pneumonia and lung fibrosis, (ii) lung biopsies from individuals with pulmonary fibrosis (PF) and (iii) control samples (control) from healthy donors reported in Habermann et al. and Bharat et al. (GSE158127, GSE135893 [35,36]). Among epithelial cells, only the basal cells expressed *PLAU* and *PLAUR* (mean and median normalized expression > 0) (Figure 1A, Table S1). In COVID-19 and PF patients, the expression of *PLAU* and *PLAUR* in these cells was significantly decreased as compared to the control (*p* < 0.0001 vs. control, Figure 1B,C). *PLAU* and *PLAUR* expression was also observed in a fraction of cells from a pro-fibrogenic KRT17+ KRT5- population in COVID-19 and PF patients (Figure 1B,C), though the overall number of *PLAU*+ and *PLAUR*+ cells in this population was still low (median normalized expression 0) (Figure 1B,C).

**Figure 2:**
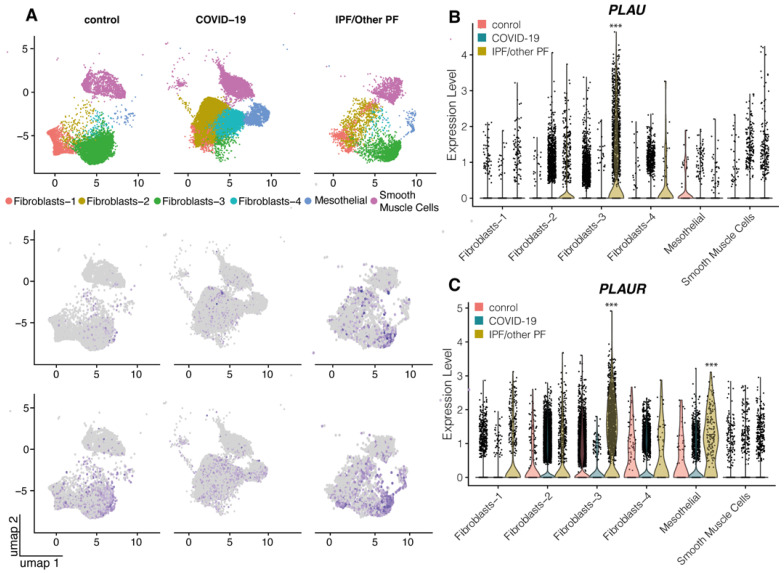
*PLAU* and *PLAUR* expression in the mesenchymal cells of control, COVID-19 and PF lung samples: (**A**) UMAP embedding of jointly analyzed single-cell transcriptomes annotated by cell type (color) and disease status. Lung tissue transcriptomes of patients with COVID-19-associated pneumonia, pulmonary fibrosis (PF) and control lung samples from healthy donors, reported in Habermann et al. and Bharat et al. (GSE158127, GSE135893 [35,36]), were analyzed. Normalized expression levels of *PLAU* (**B**) and *PLAUR* **(C)** in different cell types of mesenchymal cells; statistics were calculated only for the cell types with the median normalized expression value > 0. *** *p_adjusted_* < 0.0001 as assessed by FDR. IPF/other PF, idiopathic pulmonary fibrosis and other pulmonary fibrosis; umap, Uniform Manifold Approximation and Projection.

In control mesenchymal cells, *PLAU* expression was not normally detected (median normalized expression 0 in all cell types), and the median expression of *PLAUR* > 0 was detected only in type 4 fibroblasts (Figure 2A). Of note, type 3 fibroblasts exhibited an elevated expression of both *PLAU* and *PLAUR* in pulmonary fibrosis, while in mesothelial cells, only *PLAUR* expression was elevated (*p* < 0.0001 vs. control, Figure 2B,C). The same effect was not observed in COVID-19, and it might be accounted for the unequal representation of mesenchymal cell types in tissue samples (type 3 fibroblast population was mostly lacking in the samples obtained from COVID-19 patients) (Figure 2A).

**Figure 3:**
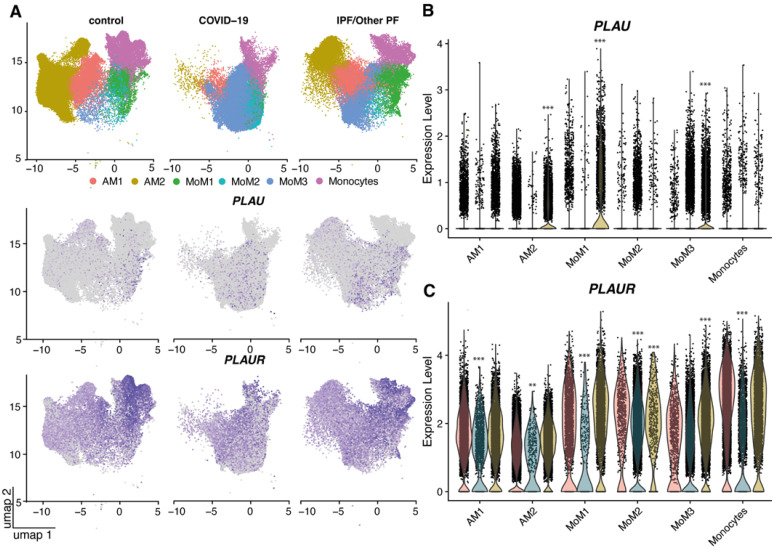
*PLAU* and *PLAUR* expression in the immune cells of control, COVID-19 and PF lung samples: (**A**) UMAP embedding of jointly analyzed single-cell transcriptomes annotated by cell type (color) and disease status. Lung tissue transcriptomes of patients with COVID-19-associated pneumonia, pulmonary fibrosis (PF) and control lung samples from healthy donors, reported in Habermann et al. and Bharat et al. (GSE158127, GSE135893 [35,36]), were analyzed. Normalized expression levels of *PLAU* (**B**) and *PLAUR* (**C**) in different types of immune cells; statistics were calculated only for cells with the median normalized expression value > 0. ns—non-significant, ** *p_adjusted_* < 0.001, *** *p_adjusted_* < 0.0001 as assessed by FDR. AM, alveolar macrophages; IPF/other PF, idiopathic pulmonary fibrosis and other pulmonary fibrosis; Mo, monocyte-derived macrophages; umap, Uniform Manifold Approximation and Projection.

The highest level of *PLAUR* expression was detected in the lung monocytes/macrophages within all the studied cells (Figure 3A,C), and its expression was significantly decreased in alveolar macrophages AM1-2, monocyte-derived macrophages MoM1-2, and monocytes of COVID-19 patients (*p* < 0.001 for AM2 and *p* < 0.0001 for other cells vs. control, Figure 3A,C). A similar decrease in *PLAUR* expression was found in MoM2 cells and monocytes of PF patients, while in MoM1 and MoM3 cells, its expression was slightly elevated. Overall, *PLAU* expression was low in all monocytes/macrophages in control and COVID-19 samples, but in PF, several AM2, MoM1 and MoM3 cells expressed *PLAU* (Figure 3B). 

Therefore, the scRNA-seq data presented here demonstrated that COVID-19 and pulmonary fibrosis displayed remarkable similarities in *PLAU*/*PLAUR* gene expression across cell lineages. *PLAUR* expression was significantly decreased in airway basal cells and in lung monocytes/macrophages, the latter being the main source of *PLAUR* expression. Along with the generally decreased *PLAU* expression in airway basal cells, specific monocytes/macrophages and fibroblasts from PF patients exhibited an upregulated *PLAU* expression. 

**Figure 4:**
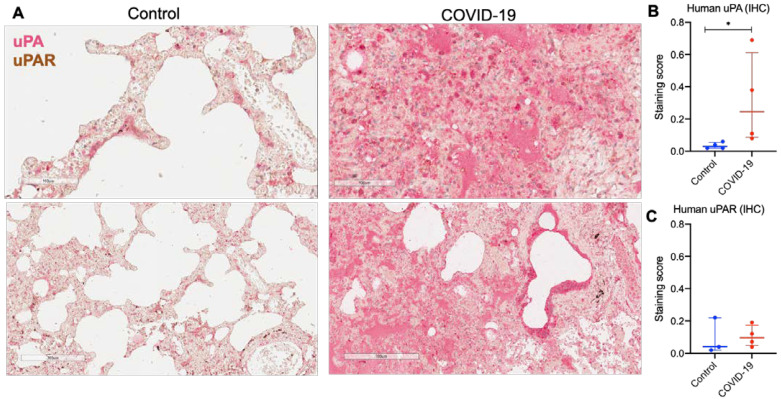
uPA and uPAR immunoreactive cells were detected in lung tissue of COVID-19 patients. (**A**) Representative sections of parenchymal tissue from COVID-19 patients and control healthy donors. uPA and uPAR were stained and revealed with ImmPACT Vector Red Substrate (uPA, pink) and DAB HRP Substrate (uPAR, brown). Scale bars—100 μm (upper panels) and 300 μm (lower panels). (**B**) uPA and (**C**) uPAR staining scores in the images of lung tissue sections of COVID-19 patients and control healthy donors. Images were analyzed using the Positive Pixel Count v9 algorithm of ImageScope (Aperio), which counts pixels of predetermined color (pink for uPA and brown for uPAR, positive pixels) and pixels related to other colors (negative pixels). A staining score was calculated as the number of positive pixels/(number of positive + negative pixels). Data are presented as median and interquartile range. * *p* < 0.05, Mann–Whitney test.

Next, we analyzed the uPA and uPAR presence in the peripheral and hilar regions of lung tissue from COVID-19 patients and the control by immunohistochemistry (Figure 4). In line with the previous data, histopathological analysis of lung autopsy samples revealed diffuse alveolar damage and features of organizing pneumonia (Figure 4A) [37]. uPA and uPAR mild immunoreactivity was detected in the alveolar epithelium of healthy control lung tissue (Figure 4A), which was consistent with scRNA-seq data that showed generally low levels of *PLAU* and *PLAUR* expression in epithelial and mesenchymal cells. The lung tissue from COVID-19 patients (*n*  =  4) was intensively stained for uPA, which was significantly higher than in the control (Mann–Whitney test, * *p* < 0.05, Figure 4A,B). uPAR staining was also detected in COVID-19 patients with no significant difference from the control (Figure 4A,C). 

Thus, uPA was excessively accumulated in the lesioned lung regions of COVID-19 patients, consistent with its reported contribution to pulmonary fibrosis [17]. However, according to scRNA-seq data, *PLAU* expression increased only in certain cell types (KRT17+ KRT5-) in COVID-19 patients; uPA protein accumulation may be linked to abnormal internalization and lysosomal degradation, directed by uPAR [38]. The significant decrease in *PLAUR* mRNA expression in basal epithelial cells and lung monocytes/macrophages (Figure 1) and its relative decrease at the background of the significantly increased amount of its ligand (Figure 4) might also contribute to the pathogenesis of pulmonary fibrosis in COVID-19. 

### 2.2. Bleomycin-Induced Pulmonary Fibrosis Was More Severe in Plaur-/- Mice

Having identified that uPA and uPAR were implicated in pulmonary fibrosis in COVID-19 and PF patients, we revealed that their expression was significantly decreased in epithelial basal cells. Next, to scrutinize the sequelae of uPA and uPAR downregulation, we used the model of pulmonary fibrosis in *Plau*- and *Plaur*-deficient mice.

**Figure 5:**
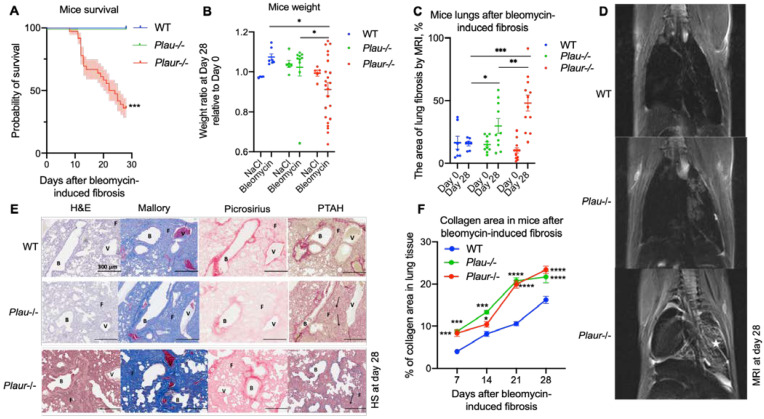
Differential progress of pulmonary fibrosis between wild-type (WT) and *Plau*-deficient (*Plau*-/-) or *Plaur*-deficient (*Plaur*-/-) mice after instillation with bleomycin. Bleomycin (3 mg/kg) was administered into WT, *Plau*-/- and *Plaur*-/- mice by intratracheal instillation at day 0. Instillation of NaCl solution was used as a control. (**A**) Kaplan–Meier survival curves, % ± SE, for WT, *Plau*-/- and *Plaur*-/- mice following intratracheal instillation of bleomycin. *** *p* < 0.001, log-rank test. (**B**) Mice were weighed before bleomycin or NaCl administration (day 0) and 28 days after. Body weight at day 28 divided by body weight day 0 (weight ratio) individual values, mean ± SEM are presented. * *p* < 0.05, 2-way ANOVA, Holm–Šídák’s test. (**C**) The % of lung tissue with fibrosis at day 0 and day 28 of bleomycin intratracheal instillation in WT, *Plau*-/- and *Plaur*-/- mice. * *p* < 0.05, ** *p* < 0.01, *** *p* < 0.001, 2-way ANOVA, Holm–Šídák’s test. (**D**) Typical MRI images obtained 28 days after bleomycin administration to WT, *Plau*-/- and *Plaur*-/- mice, the affected lung areas on the MRI images appear light, and the intact lungs appear dark. Asterisk indicates the area of severe lung damage. (**E**) Representative micrographs of 5-µm paraffin lung tissue sections stained with hematoxylin and eosin (H&E), Mallory stain (detection of total collagen), picrosirius red stain (selective detection of type I and III collagen) and phosphotungstic acid-hematoxylin stain (PTAH, detection of fibrin). Histological sections (HS) of lung tissues of WT, *Plau*-/- and *Plaur*-/- mice 28 days after bleomycin administration are presented. B, bronchus or bronchiole; F, fibrosis; V, vessel. The arrow points to collagen fibers in the pulmonary interstitium in the area of fibrosis. Scale bar—300 µm. (**F**) The analysis of collagen deposition in WT, *Plau*-/- and *Plaur*-/- mice 7, 14, 21 and 28 days after bleomycin administration by picrosirius red stain. The mean % of tissue area ± SEM with collagen deposition is presented. * *p* < 0.05, *** *p* < 0.001, **** *p* < 0.0001, 2-way ANOVA, Holm–Šídák’s test.

We used the bleomycin-induced lung injury model, which mimics the major features of pulmonary fibrosis in humans, in order to study the mechanisms of pulmonary fibrosis in wild-type (WT), *Plau*-deficient (*Plau*-/-) and *Plaur*-deficient (*Plaur*-/-) mice. Isotonic NaCl instillation was used as a control. The mice were monitored regularly for a 28-day period. Both WT and *Plau*-/- mice survived bleomycin-induced lung injury well; however, *Plaur*-/- mice demonstrated a significant mortality rate, with the overall survival being 36% at day 28 (log-rank test, *p* < 0.001, Figure 5A). Zero mortality rate was observed in the NaCl control group for all genotypes.

The increased sensitivity of *Plaur*-/- mice to bleomycin-induced fibrosis was further confirmed by measuring the mice’s weight. The weight ratio at day 28 relative to day 0 was significantly decreased in *Plaur*-/- mice after bleomycin (2-way ANOVA, Holm–Šídák’s test, *p* < 0.05, Figure 5B) as compared to the NaCl group. The worsened survival and considerable weight loss in *Plaur*-/- mice suggested more severe lung damage and uncontrolled fibrosis upon bleomycin instillation. Further, longitudinal magnetic resonance imaging (MRI) scans were used to quantitatively assess the lung damage by analyzing the difference in the % of fibrotic tissue relative to the total area at day 28 and day 0. We found that 28 days after bleomycin administration, *Plaur*-/- mice exhibited more profound lung damage than WT and *Plau*-/- mice, with 48 ± 6% of lung tissue being affected (2-way ANOVA, Holm–Šídák’s test, *p* < 0.001, *p* < 0.05, Figure 5C). In *Plau* deficient mice, the area of lung damage also increased, but less prominently: 30 ± 6% and 16 ± 2% of lung tissue were injured in *Plau*-/- and WT mice, respectively (2-way ANOVA, Holm–Šídák’s test, *p* < 0.05, Figure 5C). Figure 5D shows typical MRI images obtained 28 days after bleomycin administration to all three mice genotypes, with the affected lung tissue appearing light on the MRI images and the intact one dark. These data allowed us to propose a hypothesis that uPAR contributed more significantly to the pathogenesis of pulmonary fibrosis than uPA.

Pulmonary fibrosis is characterized by the development of aberrant and disorganized collagen deposition in the lungs. Therefore, we evaluated the uPA and uPAR- mediated effects on collagen deposition upon lung injury. Mice were lethally anesthetized 7, 14, 21 and 28 days after bleomycin administration; lungs were isolated, perfused and placed in paraffin for further slicing with a thickness of 5 μm. Tissue samples were stained using H&E, Mallory stain (to detect the total collagen), picrosirius red stain (to selectively detect type I and III collagen), and phosphotungstic acid-hematoxylin stain (PTAH, detection of fibrin) (Figure 5E). The % of the area containing type I and III collagen was estimated by analyzing the images of the picrosirius red stain. Collagen progressively accumulated after bleomycin-induced pulmonary fibrosis in all mice; collagen was intensively deposited in both *Plau*-/- and *Plaur*-/- deficient mice starting from day 7 and throughout the whole observation period (2-way ANOVA, Holm–Šídák’s test, *p* < 0.0001, Figure 5F). 

**Figure 6:**
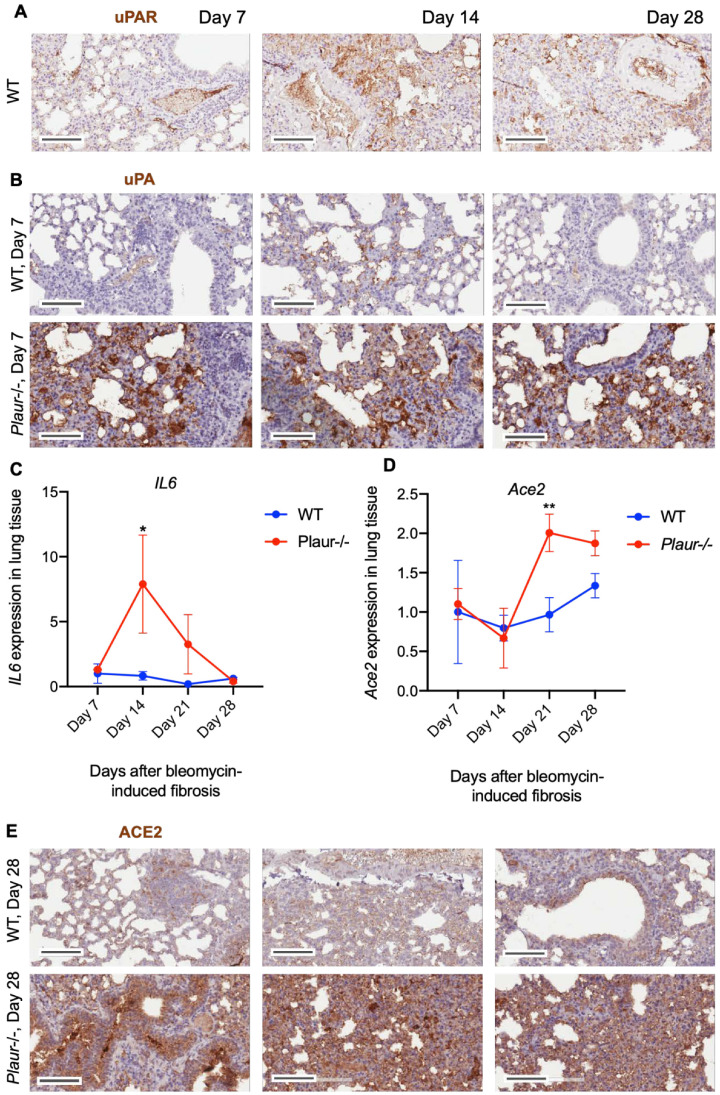
uPAR deficiency led to uPA accumulation and increased IL-6 and ACE2 expression in lung tissue following bleomycin-induced lung damage: (**A**) Representative sections of lung tissue from wild-type (WT) mice 7, 14 or 28 days after instillation with bleomycin, stained for uPAR. uPAR was revealed with DAB chromogen substrate (brown). The scale bar in images is 100 μm. (**B**) Representative sections of lung tissue from wild-type (WT) and *Plaur*-deficient (*Plaur*-/-) mice 7 days after instillation with bleomycin, stained for uPA. uPA was revealed with DAB chromogen substrate (brown), and the slides were counterstained with hematoxylin (blue). The scale bar is 100 μm. (**C**) *IL6* mRNA expression analyzed by RT-qPCR. The mRNA level was normalized to *Rpl13a* expression as a housekeeping gene; the normalization was carried out assuming the mean level of transcript in WT cells as 1. Data are presented as the mean ± SEM. * *p* < 0.05, 2-way ANOVA, Holm–Šídák’s test. (**D**) Representative sections of lung tissue from wild-type (WT) and *Plaur*-deficient (*Plaur*-/-) mice 28 days after instillation with bleomycin, stained for ACE2. ACE2 was revealed with DAB chromogen substrate (brown), and the slides were counterstained with hematoxylin (blue). The scale bar is 100 μm. (**E**) *Ace2* mRNA expression as analyzed by RT-qPCR. The mRNA level was normalized to *Rpl13a* expression as a housekeeping gene; the normalization was carried out assuming the mean level of transcript in WT cells as 1. Data are presented as the mean ± SEM. ** *p* < 0.01, 2-way ANOVA, Holm–Šídák’s test.

Thus, the absence of both uPA and uPAR led to augmented pulmonary fibrosis upon lung damage. However, uPAR deficiency exerted a more drastic effect on lung damage, weight loss and decreased survival, which confirmed its critical role in regulating fibrin deposition and cell functioning in pulmonary fibrosis. uPAR expression in WT mice resulted in no major changes throughout the observational period, serving as a valid control for further experiments (Figure 6A).

The observed dramatic effect of uPAR knockout on pulmonary fibrosis can be explained by the receptor’s role in regulating the extracellular presence and activity of its ligand uPA in the lungs. Although uPA activates fibrinolytic plasminogen, the absence of uPAR can significantly decrease uPA activity [21]. Moreover, it has been previously demonstrated that the high level of uPA exerts a prominent pro-fibrogenic effect in idiopathic pulmonary fibrosis that involves uPA-dependent IL-6 upregulation [17]. uPAR is implicated in uPA internalization and lysosomal degradation [38]; thereby, the lack of uPAR in the lung tissue of COVID-19 patients (Figure 1 and Figure 3) can contribute to excessive uPA accumulation (Figure 4), aggravating pulmonary fibrosis. This assumption was further confirmed by uPA expression analysis in *Plaur*-/- mice 7 days after bleomycin instillation: the absence of uPAR resulted in a profound accumulation of uPA in lung tissue following the bleomycin-induced damage as compared to WT mice (Figure 6B). Moreover, *IL6* mRNA expression was significantly increased in *Plaur*-/- mice 14 days after bleomycin instillation (*p* < 0.05, 2-way ANOVA, Holm–Šídák’s test, Figure 6C), confirming uPA and IL-6 in lung fibrosis to be intertwined and closely linked. In turn, IL-6 is known to upregulate ACE2, a.k.a “SARS-CoV-2 receptor” [39]; thus, we also analyzed ACE2 expression in mice lungs after bleomycin-induced pulmonary fibrosis. We found that ACE2 was upregulated in *Plaur*-/- mice at both: mRNA (*p* < 0.01, 2-way ANOVA, Holm–Šídák’s test, Figure 6D) and protein levels (Figure 6E), correlating well with higher IL-6 expression in this group (Figure 6C).

**Figure 7:**
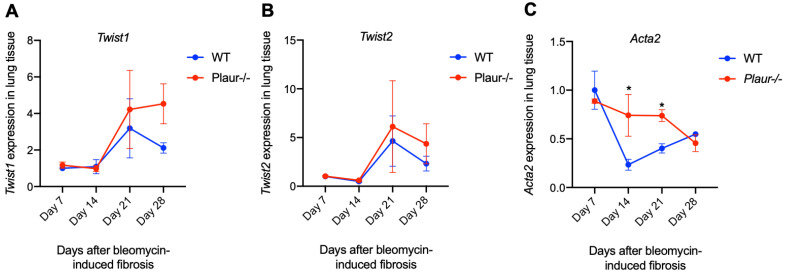
Expression of EMT markers in lung tissue samples of wild-type (WT) and *Plaur*-deficient (*Plaur*-/-) mice after instillation with bleomycin: (**A**) *Twist1*, (**B**) *Twist2*, (**C**) *Acta2* mRNA expression analyzed by RT-qPCR. The mRNA level was normalized to *Rpl13a* expression as a housekeeping gene, and the normalization was carried out assuming the mean level of transcript in WT cells as 1. Data are presented as the mean ± SEM. * *p* < 0.05, 2-way ANOVA, Holm–Šídák’s test.

EMT of alveolar epithelial cells is acknowledged to largely contribute to pulmonary fibrosis upon lung injury [40]. Since both uPAR and IL-6 considerably stimulate the EMT progression [41,42,43], we assumed that EMT induction in *Plaur*-/- mice could give a reason for more severe lung damage. We next addressed how the expression of EMT-inducing transcription factors, *Snai1*, *Snai2*, *Twist1*, *Twist2*, *Zeb1* and *Zeb2,* changed in the lungs from WT and *Plaur*-/- mice at Day 7, 14, 21 and 28 after bleomycin-induced pulmonary fibrosis. The expression of *Twist1* and *Twist2* progressively increased on Day 21 and Day 28 after bleomycin instillation (Figure 7A,B), indicating their substantial contribution to EMT in our model. We found a tendency towards their higher expression in *Plaur*-/- mice as compared to WT control, especially for *Twist1* at Day 28 (*p* > 0.05, 2-way ANOVA, Holm–Šídák’s test), though statistically non-significant.

Except for a moderate increase in *Snai1* expression at Day 21 in WT mice (Figure S1A), we found no increase in the expression of the other transcription factors after Day 7 of bleomycin-induced lung injury in WT and *Plaur*-/- mice throughout the whole experiment (Figure S1B–D). Along with this, no changes were detected in *Cdh1* (encodes for E-cadherin, an epithelial marker) and *Cdh2* expression (encodes for N-cadherin, a mesenchymal marker) (Figure S1E,F). However, a significant increase in *Acta2* expression (encodes for α-smooth muscle actin, a mesenchymal marker) was revealed in *Plaur*-/- mice on Day 14 and Day 21 of bleomycin-induced pulmonary fibrosis (2-way ANOVA, Holm–Šídák’s test, *p* < 0.05, Figure 7C). These results clearly indicated that in the absence of uPAR expression, EMT-induced pulmonary fibrosis additionally contributed to lung damage and worsened the overall outcome. We also detected no difference in *Cd4* and *Cd8* mRNA expression (Figure S1G,H).

### 2.3. uPAR Downregulation in Lung Epithelial Cells Induced Mesenchymal Phenotype

**Figure 8:**
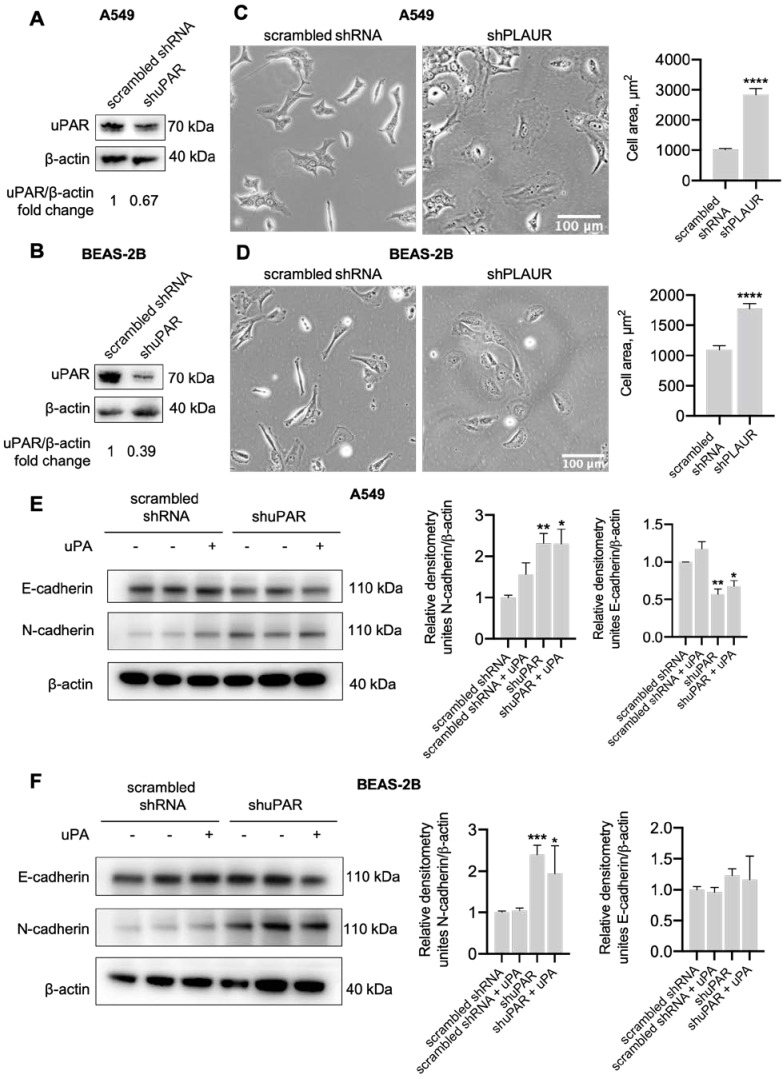
uPAR expression affected the morphology of human lung epithelial cells and changed the expression of EMT-related markers: (**A**) The level of uPAR protein expression in the obtained A549-based stable cell lines. β-actin was used as a control of protein load. Densitometric analysis of uPAR band intensities normalized to β-actin band intensities is presented relative to A549 scrambled shRNA sample. (**B**) The level of uPAR protein expression in BEAS-2B-based stable cell lines. β-actin was used as a control of protein load. Densitometric analysis of uPAR band intensities normalized to β-actin is presented relative to A549 scrambled shRNA sample. (**C**) Representative images of A549 cells with downregulated uPAR expression (shPLAUR) and respective control (scrambled shRNA), scale bar—100 µm (*left panel*). Results of cell area measurements of at least 100 cells for each cell type are presented (*right panel*). (**D**) Representative images of BEAS-2B cells with downregulated uPAR expression (shPLAUR) and respective control (scrambled shRNA), scale bar—100 µm (*left panel*). Results of cell area measurements of at least 100 cells for each cell type are presented (*right panel*). Data are presented as the mean ± SEM. **** *p* < 0.0001, *t*-test. (**E**) A549 and (**F**) BEAS-2B cells with downregulated uPAR expression (shPLAUR) and respective control (scrambled shRNA) were cultured in the absence or presence of 20 nM of uPA, and 24 h later, a Western blot analysis of E-cadherin and N-cadherin EMT markers expression was performed. β-actin was used as a control of protein load. Reproducible results of three independent experiments are presented. Densitometric analysis of band intensities for each EMT marker is presented as the mean ± SEM. * *p* < 0.05, ** *p* < 0.01, *** *p* < 0.001, ANOVA, Dunnett’s post hoc test.

We have previously shown that uPAR downregulation induced Neuro2a cell size enlargement and EMT [43]. To this end, we set out to confirm that uPAR downregulation in lung epithelial cells contributed to pulmonary fibrosis aggravation via EMT induction. In order to tackle this issue, we enrolled human A549 (adenocarcinomic human alveolar basal epithelial cells) and BEAS-2B (non-tumorigenic lung epithelial cell line) cells. We used shRNA to suppress uPAR expression (shPLAUR) and scrambled shRNA as a control. Stable cell lines expressing shPLAUR or scrambled shRNA were established after transfection and an eight-week puromycin selection. The content of uPAR at the protein level was decreased 1.5-fold and 3.6-fold in A549-shPLAUR and BEAS-2B-shPLAUR cells, respectively (Figure 8A,B).

Cells were plated in a low-density monolayer and cultured for 24 hours to allow for cell adhesion. Live-cell images were obtained and used for cell size measurement (Figure 8C,D). uPAR downregulation resulted in a pronounced phenotypic change in A549 cells (Figure 8C) and, to a lesser extent, in BEAS-2B cells. In particular, scrambled shRNA-transfected A459 cells demonstrated an epithelial-like morphology, while shPLAUR A549 cells demonstrated a rounded spread shape (Figure 8C). BEAS-2B-shPLAUR cells also presented a more rounded morphology (Figure 8D). The overall size of shPLAUR-cells was significantly larger as compared to the respective controls both for A549 (*t*-test, **** *p* < 0.0001, Figure 8C) and BEAS-2B cells (*t*-test, **** *p* < 0.0001, Figure 8D).

By using a Western blot analysis, we next examined the expression of classic markers of epithelial cell phenotype (E-cadherin) and mesenchymal phenotype (N-cadherin) in A549 and BEAS-2B cells (Figure 8E,F). The expression of E-cadherin was significantly decreased, while N-cadherin was increased in A549-shPLAUR cells (ANOVA, Dunnett’s post hoc test, *p* < 0.01, Figure 8E), pointing to EMT induction after uPAR downregulation. Administration of uPA to the cell medium for 24 h exerted no effect on EMT marker expression: A549-shPLAUR cells persistently expressed lower levels of E-cadherin and higher levels of N-cadherin compared to A549-scrambled shRNA cells (ANOVA, Dunnett’s post hoc test, *p* < 0.05, Figure 8E). Similar results were obtained in BEAS-2B cells: N-cadherin expression markedly increased in BEAS-2B-shPLAUR cells as compared to BEAS-2B-scrambled shRNA cells (ANOVA, Dunnett’s post hoc test, *p* < 0.001, Figure 8F), however, E-cadherin expression remained unchanged. Along with that, uPA treatment exerted no effect on the EMT marker expression profile in BEAS-2B-shPLAUR cells treated with uPA, where cells expressed a significantly higher level of N-cadherin as compared to BEAS-2B-scrambled shRNA cells (ANOVA, Dunnett’s post hoc test, *p* < 0.05, Figure 8F). The above results allowed us to assume that uPAR was a factor that sustained the epithelial phenotype in lung epithelial cells, while uPAR downregulation promoted the shift into a mesenchymal state with increased N-cadherin expression accompanied by substantial morphological changes. 

### 2.4. Plasminogen Administration Reversed Bleomycin-Induced Lung Fibrosis in Plaur-/- Mice

**Figure 9:**
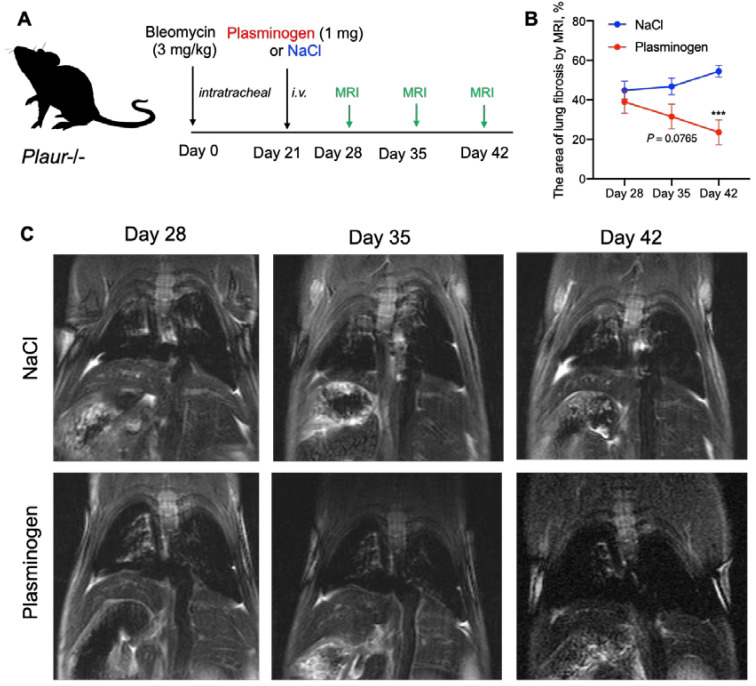
Treatment with plasminogen decreased bleomycin-induced lung fibrosis. (**A**) Overview of the experimental design. *Plaur*-/- mice were subjected to intratracheal instillation with bleomycin (3 mg/kg) at day 0. On Day 21, mice were treated with 1 mg plasminogen (*n* = 6) or isotonic NaCl (control, *n* = 7) intravenously (i.v.). MRI was performed to assess lung fibrosis on Day 28, Day 35 and Day 42 after bleomycin administration. (**B**) The % of lung tissue with fibrosis assessed by MRI at day 28, 35 or 42 of bleomycin intratracheal instillation in *Plaur*-/- mice, treated with 1 mg plasminogen or isotonic NaCl (control) intravenously at day 21. Data are presented as the mean ± SEM. *** *p* < 0.001, 2-way ANOVA, Holm–Šídák’s test. (**C**) Typical MRI images obtained 28, 35 or 42 days after bleomycin administration to *Plaur*-/- mice treated with plasminogen or NaCl (control) intravenously. For illustrative purposes, the images were taken from one and the same mouse in each group.

As we have previously shown, in the absence of uPAR, inactive uPA accumulation could lead to accelerated lung fibrosis and lower survival in lung damage conditions. Apart from uPAR, uPA can be activated by plasmin in a powerful positive feedback loop [24,25]. Thus, low levels of plasmin or its precursor plasminogen in COVID-19 patients [28,44] may contribute to profibrogenic uPA accumulation and low-level fibrinolysis. We hypothesized that the external introduction of plasminogen in the absence of uPAR might disrupt this vicious circle and reverse lung fibrosis. In order to verify this hypothesis, we induced lung fibrosis via bleomycin instillation in *Plaur*-/- mice on Day 0 and intravenously injected mouse plasminogen (1 mg) or isotonic NaCl (control) on Day 21 (Figure 9A). Lung fibrosis was monitored weekly by MRI for 3 weeks. On day 28, no differences were detected between mice assigned to different groups (Figure 9B). On Day 35, lung fibrosis had a tendency to decrease in mice treated with plasminogen (2-way ANOVA, Holm–Šídák’s test, *p* = 0.0765), while on Day 42, there was a dramatic statistically significant difference in the lung fibrosis: 23.6 ± 6.4% of the fibrotic area in plasminogen-treated mice vs. 54.5 ± 3.0% of the fibrotic area in control mice (2-way ANOVA, Holm–Šídák’s test, *p* < 0.001) (Figure 9B). Therefore, bleomycin-induced lung fibrosis progressively resolved in plasminogen-treated mice (Figure 9C). Figure S2 shows the individual % of lung fibrosis for each mouse enrolled in the experiment. Hence, the plasminogen treatment reverses lung fibrosis upon lung damage, which can serve as a promising therapeutic target for pulmonary fibrosis.

## 3. Discussion

The post-COVID-19 sequela in the form of pulmonary fibrosis is a ticking time bomb. Despite the apparent successful recovery from COVID-19, the development of pulmonary fibrosis is one of the most common complications and a serious mortality risk factor in post-COVID-19 syndrome. The early evidence suggests that even in asymptomatic individuals, pulmonary fibrosis may become one of the major long-term complications of COVID-19 [7]. Understanding the underlying mechanisms and searching for treatment options for pulmonary fibrosis is an urgent need in the post-COVID-19 era. In the current study, we found that uPAR expression was decreased in the lung tissue of COVID-19 patients, which could have led to the pro-fibrogenic accumulation of inactive uPA followed by IL-6 and ACE2 upregulation and EMT induction in lung epithelial cells. Consistently, uPAR deficiency in mice contributed to more severe pulmonary fibrosis, weight loss and poor survival, which was resolved by plasminogen treatment that substantially decreased lung fibrosis in the absence of uPAR.

Malfunction of the fibrinolytic system during COVID-19 is accompanied by the development of disseminated intravascular coagulation and increased fibrin D-dimer levels [27,45]. Plasmin is a crucial enzyme in fibrinolysis, activated by other proteolytic enzymes, including uPA. uPA was first discovered in the late 1940s in urine, so the term urokinase was coined [46]. Active uPA generates fibrinolytic plasmin from plasminogen, and this reaction is amplified by uPAR on the cell surface since the latter stimulates the conversion of pro-uPA into uPA [21,22,47]. Moreover, uPA activation is also catalyzed by plasmin itself [24,25,26]. uPAR is a mobile GPI-anchored membrane receptor that assembles the so-called uPAR-interactome, orchestrating multiple ligand-receptor interactions on the cell surface [48]. Recently, uPAR has been shown to form dimers interacting simultaneously with uPA and vitronectin [49]. This finding deepens our understanding of the partners of the urokinase system that determine its cellular effects and functions in the body.

uPA and uPAR participate in the pathogenesis of lung injury and pulmonary fibrosis [17,50], and their expression and function are subject to change in COVID-19. Bulk RNA-seq analysis of normal human bronchial epithelial cells infected with SARS-CoV2 in vitro demonstrated significant upregulation of *PLAU* and *PLAUR* [51]. Mast et al. found that both *PLAU* and *PLAUR* are significantly (~40-fold) downregulated in bronchoalveolar lavage fluid (BALF) of COVID-19 patients by bulk-transcriptomic data [52]. Another analysis of bulk BALF transcriptomes also demonstrated *PLAUR* downregulation in COVID-19 patients [51]. A more in-depth single-cell transcriptomic study of BALF by Hou et al., however, revealed that *PLAU* expression is strongly upregulated in severe COVID-19 cases as compared to healthy controls [53]. To resolve these controversies, here, we compared single-cell transcriptomic data obtained from warm postmortem lung biopsies of patients diseased from COVID-19-associated pneumonia and lung biopsies from individuals with pulmonary fibrosis and control samples from healthy donors [35,36].

COVID-19 and pulmonary fibrosis displayed important similarities in *PLAU*/*PLAUR* gene expression across epithelial and monocyte/macrophage cell lineages. Our findings demonstrate that the lung monocytes/macrophages serve as a main source of *PLAUR* expression, which was significantly decreased in COVID-19 and PF (Figure 3A,C). In contrast, *PLAU* expression was overall low in all monocyte/macrophage cell types in control lungs, but under the PF condition, specific alveolar and monocyte-derived macrophages appeared to express *PLAU* (Figure 3B). Among all epithelial cells, only airway basal cells were detected to persistently express *PLAU* and *PLAUR,* and their expression was significantly decreased in COVID-19 and PF patients (Figure 1). Additionally, in both conditions, a pro-fibrogenic epithelial cell population of KRT17+ KRT5- cells emerged, with some cells expressing detectable *PLAU* and *PLAUR* levels (Figure 1). Pulmonary fibrosis was associated with the elevated expression of *PLAU* and *PLAUR* in mesenchymal cells: *PLAU* in type 3 fibroblasts, *PLAUR* in type 3 fibroblasts and mesothelial cells (Figure 2A). We also confirmed that the changes in *PLAU* mRNA expression reflected in a relative change in its protein level: the lung tissues from COVID-19-deceased patients contained excessive levels of uPA as compared to the control (Figure 4). Therefore, *PLAUR* expression was significantly decreased in epithelial and monocyte/macrophage cells from COVID-19 patients as compared to control samples. It has been previously reported that uPAR expression is critical not only for uPA activation on the cell surface [19,20,21,22] but also for its controlled internalization and lysosomal degradation with in the complex with its endogenous inhibitor PAI-1 [38,43,54,55]. The decrease in uPAR expression can perturb both processes and result in the excessive accumulation of inactive uPA in the lungs, observed in COVID-19 patients (Figure 4). This assumption was verified by a comparative analysis of uPA in the lungs of WT and *Plaur*-deficient mice 7 days after bleomycin-induced pulmonary fibrosis revealing excessive uPA accumulation in the absence of *Plaur* (Figure 6B). We previously showed that the decreased PAI-1 level is a significant risk factor for COVID-19-related death in severely ill patients [27], reflecting the exhaustion of compensatory mechanisms for hyperactivation of the plasminogen activator system in COVID-19. We also found that the increased IL-17 level is a mortality risk factor in COVID-19 patients [27]. In addition, IL-17 was previously shown to upregulate uPA expression [56].

uPA functionality is double-edged as it has the well-acknowledged fibrinolytic activity as well as the pro-fibrotic one in case of excessive and/or inactive uPA accumulation. In blood vessels, the application of high doses of uPA causes negative vascular remodeling, reduction in lumen size and increased number of smooth muscle cells [57,58]. In addition, uPA knockout in mice suppressed cardiac fibrosis in the model of transverse aortic banding and coxsackievirus-B3-induced myocarditis [57,58]. Moreover, in idiopathic pulmonary fibrosis, uPA exerts a strong pro-fibrotic effect that involves uPA-dependent IL-6 upregulation [17]. In the absence of uPAR, uPA activity decreased and renal fibrosis in obstructive nephropathy was aggravated [59].

Previous histopathological studies demonstrated increased fibrosis in bleomycin- and silica particles-induced pulmonary fibrosis in *Plau*-/-, but not in *Plaur*-/- mice [60,61]. However, in these studies, the mice’s survival or weight loss, as well as lung fibrosis by MRI analysis, were beyond the scope. We compared in detail how pulmonary fibrosis proceeded in *Plau*- and *Plaur*-deficient mice in a model of bleomycin-induced lung injury (Figure 5). The % of the collagen-positive area of the lung tissue, as revealed by histological staining, was the same among *Plau*- and *Plaur*-deficient mice and was enlarged compared to WT mice (Figure 5E,F). However, *Plaur*-/- mice experienced a significantly higher mortality rate compared to WT and *Plau*-/- mice, with the overall survival rate being 36% at day 28 in *Plaur*-/- mice and 100% in WT and *Plau*-/- mice (Figure 5A). It is known that pulmonary fibrosis induces considerable weight loss, which correlates with lower survival [62]; consistently, we showed that *Plaur*-/- mice had a significant weight loss upon bleomycin-induced pulmonary fibrosis compared to WT and *Plau*-/- mice (Figure 5B). Finally, as opposed to WT and *Plau*-/- mice, *Plaur*-/- mice exhibited more severe lung damage and fibrosis on MRI scans (Figure 5C,D). These data allowed us to draw the following conclusion: uPAR downregulation, complemented by inactive uPA accumulation, led to augmented pulmonary fibrosis upon lung damage.

Furthermore, we confirmed that the worsened pulmonary fibrosis in *Plaur*-/- mice was accompanied by IL-6 upregulation (Figure 6C), which was in agreement with the previously established link between uPA and IL-6 in lung fibrosis [17]. IL-6, in turn, is known to upregulate ACE2, the “SARS-CoV-2 receptor” [39], and, indeed, ACE2 was upregulated in *Plaur*-/- mice lungs after bleomycin-induced lung injury (Figure 6D,E). Our findings indicate that uPAR downregulation and the increased uPA levels in lung tissue of COVID-19 patients may substantially aggravate pulmonary fibrosis and lead to IL-6 and ACE2 upregulation. High plasma levels of IL-6 are strongly associated with lower survival in COVID-19 patients [63,64,65]. ACE2 enhances the interaction of SARS-CoV-2 with epithelial cells, exacerbating the disease and COVID-19 prognosis at large [66,67,68].

As reported earlier, both uPAR and IL-6 regulate EMT progression [41,42,43], a process that actively contributes to pulmonary fibrosis upon lung injury [40]. Here we demonstrate that significantly more severe lung damage in *Plaur*-/- mice can also result from EMT induction: the expression of α-smooth muscle actin (*Acta2*), a mesenchymal marker, was significantly increased in *Plaur*-/- mice compared to WT mice (Figure 7C). Modeling uPAR downregulation in lung epithelial cell lines A549 and BEAS-2B showed similar results: uPAR downregulation increased N-cadherin expression (a mesenchymal marker) (Figure 8E,F), indicating the EMT induction. We also observed characteristic morphologic changes in lung epithelial cells with downregulated uPAR, which exhibited a more rounded and spread morphology (Figure 8C,D), in accordance with the previous published data on neuroblastoma, melanoma and colon cancer cells [43,69,70]. Our current results suggest that uPAR is a factor that sustains the epithelial phenotype in lung epithelial cells, and the absence of uPAR in lung tissue may additionally stimulate EMT induced by pulmonary fibrosis, worsening lung damage and overall outcome. Moreover, our data correlate well with the previous report on the role of uPAR in alveolar type II epithelial cells EMT, which showed that upon exposure to bleomycin, uPAR-deficient alveolar type II lung epithelial cells undergo increased EMT [50]. Coming full circle, we have previously shown that uPAR downregulation induces uPA- and IL-6-dependent EMT in Neuro2a cells [50].

**Figure 10:**
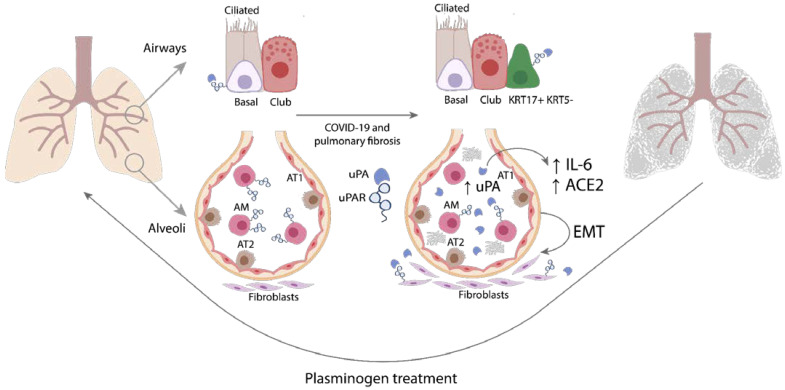
The putative scheme of COVID-19-induced pulmonary fibrosis regulation by uPA/uPAR system. In control conditions, only the epithelial basal airway cells express uPA and uPAR, while lung monocytes/macrophages express a considerable amount of uPAR. COVID-19 and pulmonary fibrosis result in uPA and uPAR downregulation in basal cells, uPAR downregulation in lung monocytes/macrophages, and the appearance of pro-fibrogenic KRT17+ KRT5- cells and fibroblasts that express both uPA and uPAR. Additionally, pulmonary fibrosis is associated with the emergence of some alveolar and monocyte-derived macrophages that express uPA. The consequences of uPAR downregulation involve uPA accumulation due to the defects in its internalization and lysosomal degradation. Pro-fibrogenic uPA accumulation in the absence of uPAR leads to IL-6 and ACE2 upregulation and EMT. Excessive lung fibrosis induced by low uPAR levels and the accumulation of inactive uPA can be resolved by plasminogen treatment. AT1 and AT2, alveolar type I and type II cells; AM, alveolar macrophages; EMT, epithelial–mesenchymal transition.

Apart from uPAR, uPA can be activated by plasmin in a powerful positive feedback loop [24,25]; hence, excessive lung fibrosis, associated with low uPAR levels and the accumulation of inactive uPA, can be resolved by plasmin or its precursor plasminogen. For the first time, we demonstrated that the introduction of the external plasminogen could indeed disrupt this vicious circle and reverse lung fibrosis in the absence of uPAR: the intravenous injection of 1 mg of plasminogen on the 21st day of bleomycin-induced fibrosis in *Plaur*-/- mice resulted in a 2-fold reduction in the fibrosis area in the lungs as compared to the non-treated mice by day 42 (Figure 9). Although initially, plasminogen is not efficiently activated in the presence of inactive uPA, the reaction can still be initiated by the low intrinsic catalytic activity of pro-uPA. Following the lag-phase, plasmin can be exponentially generated through the feedback loop when pro-uPA is activated by the already generated plasmin [26]. The described mechanism can provide a rationale for the effect of plasminogen treatment on pulmonary fibrosis resolution being detected three weeks after its injection (Figure 9). Low blood levels of plasmin and its precursor plasminogen correlate with a high risk for mortality and are associated with IL-6 upregulation in COVID-19 patients [27,28,44], reflecting its potential contribution to profibrogenic uPA accumulation and low-level fibrinolysis. This very role of plasmin is evidenced by the suppression of plasmin-mediated fibrinolysis leading to the accumulation of airspace fibrin in idiopathic pulmonary fibrosis [18]. In clinics, plasminogen treatment (Ryplazim) was already approved by FDA in 2021 for congenital plasminogen deficiency [71]. This provides a promising therapeutic avenue for plasmin-based treatment of various types of pulmonary fibrosis, which should be explored further (Figure 10).

There are some limitations to this study. Firstly, human lung tissue samples from only four COVID-19-deceased patients were analyzed for uPA and uPAR expression, and a bigger sample analysis could corroborate our conclusions. However, the obtained results showed good reproducibility among the samples, and we used these data in combination with the analysis of the single-cell RNA-seq of lung tissue samples from an independent cohort of COVID-19-deceased patients reported by Bharat et al. [35]. Secondly, the data on EMT TFs *Twist1* and *Twist2* in *Plaur*-deficient mice lack statistical significance due to the small animal cohort size; they are, however, in agreement with the previously obtained data [50] and were confirmed by *Acta2* expression analysis and in cell line models. Despite these limitations, the current study revealed an important and potentially targetable mechanism to prevent COVID-19-related pulmonary fibrosis.

## 4. Materials and Methods

### 4.1. Human Lung Tissue Samples

We analyzed pulmonary autopsy specimens from 15 unvaccinated patients who died from respiratory failure caused by SARS-CoV-2 infection as confirmed by PCR and compared them with lungs from 11 healthy donors who died from car accidents. The patients were hospitalized in the National Medical Research Center of Cardiology, named after Academician E.I. Chazov (Moscow, Russia), from April to July 2021. Demographic, clinical and laboratory data were recorded at admission. The study was approved by and conducted according to the requirements of the ethics committee at the NMRC of Cardiology, named after Academician E.I. Chazov (Moscow, Russia). There was no commercial support for this study. 

### 4.2. Animals

Weight- and age-matched adult C57BL/6N wild-type mice (WT, “Pushchino”, Pushchino, Russia), *Plau*-/- mice (originally derived from C57/Bl6 mice by a group of Carmeliet from the FIRC Institute of Molecular Oncology, Milan, Italy [72]) and *Plaur*-/- mice (originally derived from C57Bl6/SV129 mice by Flanders Institute for Biotechnology, Gent, Belgium) were enrolled in the study. Mice were kept in cages by three and maintained under a standard 12 h light cycle, with temperature 20–24 °C and humidity 35–70%. Water and food were available ad libitum. Animal housing was performed in accordance with the European Convention for the Protection of Vertebrate Animals used for Experimental and other Scientific Purposes ETS №123. Careful consideration was given to the number of animals: a cohort of 143 animals, 65 males and 78 females, 8–20 weeks was enrolled, the minimum number required to obtain valid results. Particular effort was made to minimize the animals’ pain and distress. For the following series of experiments, mice were randomly divided into two groups. The experimental procedures were conducted in accordance with Directive 2010/63/EU of the European Parliament and the Council of 22 September 2010 on the protection of animals used for scientific purposes. All manipulations with animals were approved by the local ethical committee in accordance with the in-house requirements of the Commission on Bioethics of the Lomonosov Moscow State University (license numbers 3.3 and 3.4).

### 4.3. Bleomycin-Induced Pulmonary Fibrosis in Mice

Bleomycin-induced lung injury was used to model pulmonary fibrosis in mice, as described previously [73]. Briefly, bleomycin (Nippon Kayaku, Tokyo, Japan, 3 mg/kg) was administered into WT (*n* = 19), *Plau*-/- (*n* = 22) and *Plaur*-/- (*n* = 53) mice by intratracheal instillation, administration of isotonic saline was used as a control (WT *n* = 16; *Plau*-/- *n* = 16, and *Plaur*-/- *n* = 17). Bleomycin administration was accompanied by isoflurane anesthesia via inhalation of an air mixture containing 2–4% of isoflurane (Laboratorios Karizoo, S.A., Barcelona, Spain) and 93% of oxygen (V3000 vaporizer, Parkland Scientific, Coral Springs, FL, USA, with Nuvo Lite 525 oxygen concentrator, Nidek medical products, Birmingham, AL, USA). The administration of bleomycin was counted as day 0. The mice were then observed weekly for weight changes, survival and lung fibrosis evaluated by MRI. 

### 4.4. Bolus Administration of Plasminogen to Mice

On the 21st day after bleomycin administration, *Plaur-/*- mice with signs of fibrosis based on MRI data were selected. On the same day, mice were randomly divided into two groups, the first group (*n* = 6) received an i.v. bolus of purified mouse plasminogen (Innovative Research, IMSPLG1MG, Novi, MI, USA), the second group received NaCl (*n* = 7). A single injection of plasminogen was performed. MRI images were obtained 28, 35 or 42 days after bleomycin administration for both groups.

### 4.5. MRI for Noninvasive Evaluation of Lung Fibrosis

MRI images were acquired using a ClinScan 7T tomograph (Bruker Biospin, Billerica, MA, USA) as previously described [73]. Mice were anesthetized by inhalation of an air mixture containing 2–4% of isoflurane (Laboratorios Karizoo, S.A., Barcelona, Spain) and approximately 93% of oxygen (V3000 vaporizer, Parkland Scientific, Coral Springs, FL, USA, with Nuvo Lite 525 oxygen concentrator, Nidek medical products, Birmingham, AL, USA). An MR-compatible Model 1025 Small Animal Monitoring and Gating System (Small Animal Instruments, Inc., Brookhaven, NY, USA) was used to synchronize the exposure with the respiration rate. Lung magnetic resonance imaging data were obtained in fat-suppressed T2-weighted turbo-spin-echo sequences with the following parameters: TR = 1175 ms, TE = 55 ms, echo train length = 8, FOV 42 × 60 mm, base resolution 216 × 384. 

In order to evaluate lung fibrosis, we used the MRI frontal projection. The image covering the lungs with the most affected area avoiding the heart and large pulmonary vessels, was manually selected. Imaging data were quantified using ImageJ (National Institutes of Health, Bethesda, MA, USA). 

The lung fibrosis was scored by applying the following algorithm:

1. The background signal of the image was adjusted (the brightness level was set so that 99.5% of pixels outside the mouse body were dark);
2. Regions of interest (ROIs) covering the lung tissue but avoiding the heart and large pulmonary vessels were manually selected;
3. The percentage of dark (“healthy”) pixels in the ROI based on the pre-set background brightness was calculated. The resulting value was derived by subtracting the percentage of dark pixels from 100%, which represents the proportion of fibrotic tissue in the lungs.

### 4.6. Mouse Lung Isolation and Sample Collection

Mice were euthanized with the overdosing of a mixture of zoletil (Zoletil 100 Virbac, Carros, France, 30 mg/mL) with xylazine (Xyla, Interchemie werken “De Adelaar” Eesti AS, Viimsi vald, Estonia, 3 mg/mL) in sterile saline. Immediately lungs were perfused using a 3mL syringe with a 22 g needle to inject NaCl into the right ventricle of the heart until the lungs turned white. Further, lungs were harvested and divided into two groups for morphometric evaluation and RNA analysis. Specimens destined for morphometric analysis were embedded in paraffin; lung specimens for RNA analysis were immediately flash-frozen in liquid nitrogen for qPCR.

### 4.7. Immunohistochemistry, Microscopy and Morphometric Analysis

Five μm-thick formalin-fixed, paraffin-embedded sections of human parenchymal lung tissues were deparaffinized in xylene and rehydrated in a gradually decreasing concentration of methanol (100%, 95% and 70%); antigen retrieval was carried out with Trilogy buffer (Sigma-Aldrich, Merck Group, Darmstadt, Germany) according to the manufacturer’s protocol. The slides were then rinsed 2 times with PBS for 5 min. Endogenous peroxidase was blocked using 3% hydrogen peroxide solution for 20 min; the slides were then washed 2 times with PBS for 5 min. Nonspecific binding was blocked with 1% BSA/PBS blocking solution for 30 min at room temperature. The slides were incubated with primary antibodies against uPA (Biolegend, San Diego, CA, USA cat #369002), uPAR (Abcam, Cambridge, UK, ab103791) or ACE2 (CloudClone, Wuhan, China, PAB886Mu01); diluted 1:100 in blocking solution for 1 h at room temperature; and washed 2 times with PBS for 5 min. Further, slides were incubated with secondary ready-to-use peroxidase-conjugated Real EnVision antibodies (Dako, Carpinteria, CA, USA) for 30 min at room temperature for single staining or with AP horse anti-mouse IgG (ImmPRESS HRP/ AP Polymer System, Vector laboratories, Burlingame, CA, USA) for double staining for 30 min at room temperature, followed by 3 washes with PBS for 5 min. In the case of single staining, HiDef Detection™ HRP Polymer Detector (HiDef Detection HRP Polymer system, Cell marque, Rocklin, CA, USA) was also applied for 10 min at room temperature, followed by a rinse with PBS for 5 min. For single staining, DAB chromogen substrate (Dako, Carpinteria, CA, USA) was added for 2 min, and the reaction was stopped in distilled water. For double staining, ImmPACT DAB EqV Substrate incubation was followed by ImmPACT Vector Red Substrate (ImmPRESS HRP/AP Polymer System, Vector laboratories, Burlingame, CA, USA) for 2 min, and the reaction was stopped in distilled water. Finally, the slides were dehydrated in graded methanol (70%, 95%, 100%) and xylene baths and mounted with Cytoseal™ 60 (Thermo Fisher Scientific, Waltham, MA, USA).

The slides were scanned using Aperio ImageScope (v12.4.3.5008, Leica Microsystems GmbH, Wetzlar, Germany). Quality control of the scanned images and all further analysis were performed using Aperio ImageScope. All images were analyzed with the exclusion of internal contents of blood vessels using the negative pen tool to eliminate nonspecific detection in this area. Slides were analyzed using the Positive Pixel Count v9 algorithm, which counts pixels of a predetermined color, intensity and saturation. The algorithm input parameters were initially set to obtain the identification of pixels related to brown or pink (positive pixels) and the identification of pixels related to other colors (negative pixels). The algorithm output is composed of the number of positive pixels (Np) and the number of negative pixels (Nn). A staining score is then defined and calculated by the algorithm as Np/(Np+Nn).

### 4.8. Histopathologic Evaluation of Lung Fibrosis

Serial sections of paraffin-embedded mouse lung tissues were histologically stained for (1) total collagen using Mallory stain, (2) collagen types I and III using picrosirius red stain, (3) fibrin using phosphotungstic acid–haematoxylin stain, and counterstained with hematoxylin and eosin (H&E). The resulting samples were imaged with Aperio ImageScope. The area of tissue containing type I and III collagen, as revealed by the picrosirius red stain, was evaluated using the ImageJ image analysis and processed with software. In order to accomplish this, a color photograph of a section obtained with a Leica light microscope was divided into three layers (image, color, color deconvolution—H AEC); on the selected layer, the total cut area and the area of positive bright color were determined (image, adjust, threshold). Next, we calculated the percentage of the positive collagen staining as related to the total area of the analyzed sample. For each time point, lung sections from at least three animals of each genotype were analyzed.

### 4.9. Plasmid Cloning

shRNA targeting the human *PLAUR* gene was designed using the GPP Web Portal (the RNAi Consortium, https://portals.broadinstitute.org/gpp/public/, accessed on 15 December 2021). Scrambled shRNA sequence was imported from Addgene plasmid #1864 [74]. Single-stranded DNA oligos encoding shRNA sequences were purchased from Eurofins Genomics, Germany (Table S2), annealed, phosphorylated using T4 polynucleotide kinase (Thermo Fisher Scientific, Waltham, MA, USA, #EK0031) and ligated into the pLKO.1 plasmid with puromycin resistance gene (Addgene, Watertown, MA, USA, #8453, [75]) using T4 DNA ligase (Thermo Fisher Scientific, Waltham, MA, USA, #EL0011). The plasmid was digested by the AgeI and EcoRI restriction enzymes (New England Biolabs, Ipswich, MA, USA, #R0552S, #R0101M) and dephosphorylated using shrimp alkaline phosphatase (Fermentas, Waltham, MA, USA, # EF0511) prior to ligation. The sequence of resulting plasmids encoding *PLAUR* shRNA (pLKO.1-shPLAUR) or scrambled shRNA (pLKO.1-scrambled-shRNA) was confirmed using Sanger sequencing. 

### 4.10. Cell Culture

Human BEAS-2B (non-tumorigenic lung epithelial cell line) and A549 (adenocarcinomic human alveolar basal epithelial cells) cells were used to analyze the role of uPAR in lung fibrosis in vitro. Cells were cultured in a complete medium: DMEM, 10% FBS, 1 × antibiotic-antimycotic solution (all from Gibco, New York, NY, USA), 5%CO2, 37 °C. Cells were plated at a concentration of 1 × 10^5^ cells/mL. BEAS-2B and A549 cells with downregulated uPAR expression and respective controls were obtained by transfecting cells with pLKO.1-shPLAUR or pLKO.1-scrambled-shRNA plasmids, respectively, using Viafect (Promega, Madison, WI, USA) transfection reagent. Stable cell lines were obtained by culturing cells in a complete medium with the addition of 2.5 µg/mL Puromycin for at least 8 weeks (Sigma-Aldrich, Burlington, MA, USA). Analysis of the uPAR expression level in the obtained stable cell lines is presented in Figure 6A,B.

### 4.11. Cell Size Measurement

BEAS-2B and A549 cells with downregulated uPAR expression and respective controls were plated into T-75 flasks at a concentration of 2  ×  10^6^/flask. After 48 h, cells were imaged using Invitrogen EVOS XL Core imaging system (Thermo Fisher Scientific, Waltham, MA, USA), 20× resolution. The cell size (cell area) of isolated cells was evaluated using the ImageJ software (National Institutes of Health, Bethesda, MA, USA) as previously described [43]. The size of at least 100 cells was measured for each cell type.

### 4.12. RNA Isolation, Reverse Transcription and qPCR

Total RNA was isolated from the frozen lung tissue using TRIzol Reagent (Evrogen, Moscow, Russia) and from cultured cells using the NucleoSpin RNA kit (Macherey-Nagel, Düren, Germany) according to the manufacturer’s instructions. The quantity and quality of total RNA were evaluated using NanoDrop 1000 Spectrophotometer (Thermo Fisher Scientific, Waltham, MA, USA). Total RNA (1 μg) was reverse-transcribed using oligo(dT) and random (dN)_10_ primers with MMLV RT kit (Evrogen, Moscow, Russia) or Maxima™ H Minus cDNA Synthesis Master Mix (Thermo Fisher Scientific, Waltham, MA, USA). PCR was carried out using qPCRmix-HS SYBR (Evrogen, Moscow, Russia) on a CFX96 Touch Real-Time PCR Detection System (BioRad, Hercules, CA, United States). The murine cDNA primers (Table S3) were obtained from Evrogen (Moscow, Russia). The thermal cycling program was as follows: a 5 min denaturing step at 95 °C followed by 40 amplification cycles consisting of 15 s of denaturation at 95 °C, 15 s of annealing at 62 °C and 20 s of extension at 72 °C. qPCRs for each sample were performed in triplicates (technical replicates). A relative transcript level was calculated using the Pfaffl method [76] with *Rpl13a* as a reference gene; the mean level of each transcript in the WT group (7 days) was set as 1. The analysis of primer efficiency was performed by plotting the cycle threshold value (Ct) against the serial 1:10 dilution of the cDNA sample using the equation $E=10^{-\frac{1}{the\;slope\;value}}\;$(Figure S3).

### 4.13. Protein Extraction, SDS-PAGE Electrophoresis and Western Blot

Cells were collected by centrifugation for 10 min at 800g. Cell pellets were resuspended in NETN buffer (150M NaCl, 1mM EDTA, pH 7.5 50 mM Tris, 0.5% NP 40, protease and phosphatase inhibitor cocktails), sonicated for 10 s at 30%, incubated on ice for 30 min, and centrifuged at 4 °C at 12,000× *g* for 10 min. The supernatant was transferred into a new pre-cooled microcentrifuge tube, and the cell pellet was discarded. Protein quantification was performed using the Pierce™ BCA Protein Assay Kit (Thermo Fisher Scientific, Waltham, MA, USA) on the NanoDrop 2000C (Thermo Fisher Scientific, Waltham, MA, USA). After measuring the concentration, cell lysates used for Western blot were supplemented with Laemmli buffer (LDS) and 0.1M DTT, then heated at 95 °C for 10 min.

Protein samples (20 μg) and prestained molecular weight markers (PageRuler™ Prestained Plus Protein Ladder, Thermo Fisher Scientific, Waltham, MA, USA) were resolved on 12-well precast SDS-PAGE gels (4–12%) (NuPage, Carlsbad, CA, USA) in MOPS Running Buffer (NuPage, Carlsbad, CA, USA). Proteins were transferred onto 0.45 μm PVDF membrane (GE Healthcare, Chicago, IL, USA) in a transfer buffer (0.025M Tris, 0.192M Glycine, 20% ethanol) at 90V at + 4 °C for 2 h. Nonspecific binding was blocked in 5% non-fat dried milk in Tris-buffered saline, 0.1% Tween-20 (TBST) for 1h at room temperature.

Proteins were probed at +4 °C overnight with the following primary antibodies: rabbit anti-uPAR (1:500, Abcam, Cambridge, UK, ab103791), rabbit anti-N-cadherin (1:1000, Abcam, ab76011), rabbit anti-E-cadherin (1:500, Abcam, Cambridge, UK, ab40772), mouse anti-β-actin (1:1000, control of protein load, Santa Cruz, Biotechnology, Dallas, TX, USA, cat. # sc-81178). Membranes were washed with TBST and incubated with the appropriate peroxidase-conjugated secondary antibodies (goat anti-mouse IgG-HRP (Santa Cruz Biotechnology, Dallas, TX, USA, cat. # sc-2005), donkey anti-rabbit IgG-HRP (Santa Cruz Biotechnology, Dallas, TX, USA, cat. # sc-2313)) in 1:1000 dilution for 2 h at room temperature, and followed by washing in TBST. Proteins were visualized using Immobilon Western Chemiluminescent HRP Substrate (Millipore, Burlington, MA, USA) and ImageQuant LAS 4000 mini (GE Healthcare, Chicago, IL, USA) for Western blot imaging and analysis. Densitometric analysis of blots at non-saturating exposures was performed using ImageJ. The original uncropped Western blot images are presented in Figure S4.

### 4.14. Transcriptome Analysis

Publicly available transcriptomic datasets were used in the study. Single-cell RNA-sequencing (scRNA-seq) data were obtained from two warm postmortem lung biopsies of patients diseased from COVID-19-associated pneumonia (GSE158127), 10 lung biopsies from individuals with pulmonary fibrosis (GSE135893) and 12 control samples from healthy donors from both datasets. The machine learning-integrated dataset, including all samples, was downloaded from [35]. In the integrated dataset, the cell type annotations from Habermann et al. [36] and Bharat et al. [35] were used to separate cells by lineage, and the clustering was performed independently for cell lineages using latent space coordinates as input (see [35] for details). The data were processed using the Seurat R package (https://satijalab.org/seurat/, accessed on 9 March 2022) [77]. The code for obtaining the images used in the article is available at https://github.com/AnnaK135/Shmakova2022_fibrocovid (accessed on 4 July 2022).

### 4.15. Data and Statistical Analysis 

Data were analyzed using GraphPad Prism 9 software (GraphPad Software Inc., San Diego, CA, USA). The unit of analysis was a single animal or single human lung biopsy. Differences in the uPA and uPAR expression in COVID-19 patients vs. healthy controls analyzed by immunohistochemistry were determined using Mann–Whitney test. Mice survival was compared using a log-rank test. Student’s unpaired *t*-tests were used to compare cell sizes between two groups. One-way analysis of variance (ANOVA), followed by Dunnett’s post hoc test, was used to determine differences between more than two groups that involved one factor. Two-way ANOVA, followed by Holm–Šídák’s post hoc test, was used to determine differences between two or more groups that involved two factors. Data are presented as a mean ± standard error of the mean (SEM). The level of significance was set at *p* < 0.05.

## 5. Conclusions

In conclusion, pulmonary fibrosis is a serious complication of COVID-19 with no definitive treatment. The data presented here demonstrate that COVID-19 and pulmonary fibrosis may drive molecular responses of the plasminogen activator system, aggravating fibrosis and exacerbating the outcome. This pathogenic effect appears to be mediated by the decrease in uPAR expression in the specific lung cells of COVID-19 patients, which may lead to pro-fibrogenic accumulation of inactive uPA followed by IL-6 and ACE2 upregulation and EMT induction (Figure 10). Consistent with this, uPAR deficiency in mice led to more severe pulmonary fibrosis, weight loss and poor survival. This pathogenic effect can be resolved by plasminogen treatment, which substantially decreases lung fibrosis in the absence of uPAR. Further investigation is needed to determine the therapeutic potential of plasminogen in the treatment of post-COVID-19 pulmonary fibrosis.

## Data Availability

All data generated and/or analyzed during this study are included in this published article [and the Supplementary Information files].

## Supplementary Materials

The following supporting information can be downloaded at: https://www.mdpi.com/article/10.3390/ijms24021382/s1. References [78,79] are cited in the supplementary materials.
