# Supplementary material for: Urokinase System in Pathogenesis of Pulmonary Fibrosis: A Hidden Threat of COVID-19
Source: Int J Mol Sci. 2023 Jan 10;24(2):1382. doi: 10.3390/ijms24021382 (PMC9867169; doi:10.3390/ijms24021382)
Supplement: Supplementary file 1 [file ijms-24-01382-s001.zip › ijms-2083122-supplementary/ijms-2083122 Supplementary Final.pdf]

Supplementary figures

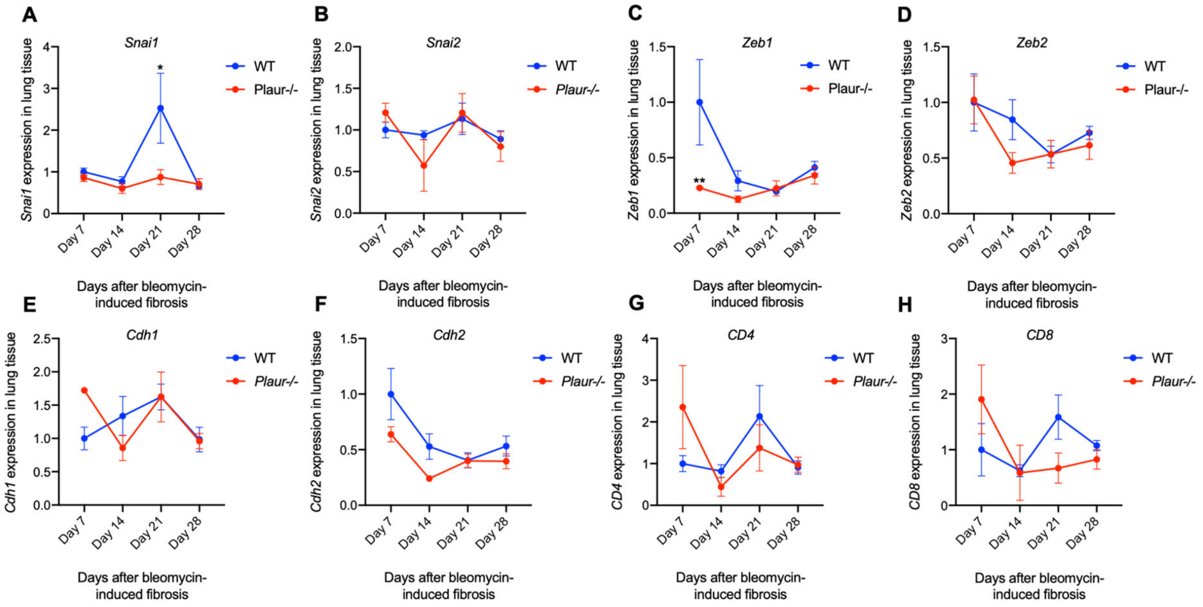

Figure S1. Expression of EMT and T lymphocyte markers in lung tissue samples of wild-type (WT) and *Plaur*-deficient (*Plaur*-/-) mice after instillation with bleomycin. (A) *Snai1*, (B) *Twist2*, (C) *Zeb1*, (D) *Zeb2*, (E) *Cdh1*, (F) *Cdh2*, (G) *CD4*, (H) *CD8* mRNA expression analyzed by RT-qPCR. The mRNA level was normalized to *Rpl13a* expression as a housekeeping gene, the normalization was carried out assuming the mean level of transcript in WT cells as 1. Data are presented as the mean ± SEM. * $p < 0.05$, 2-way ANOVA, Holm-Šídák's test.

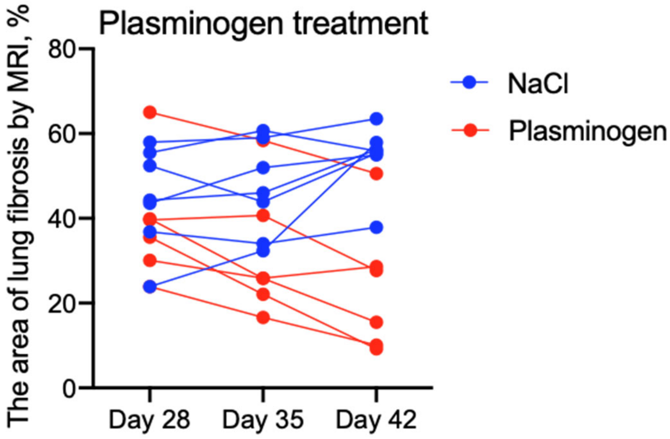

Figure S2. Individual values of the % of lung tissue with fibrosis at day 28, 35 or 42 of bleomycin intratracheal instillation in *Plaur*-/- mice, treated with 1 mg plasminogen or isotonic NaCl (control) intravenously at day 21.

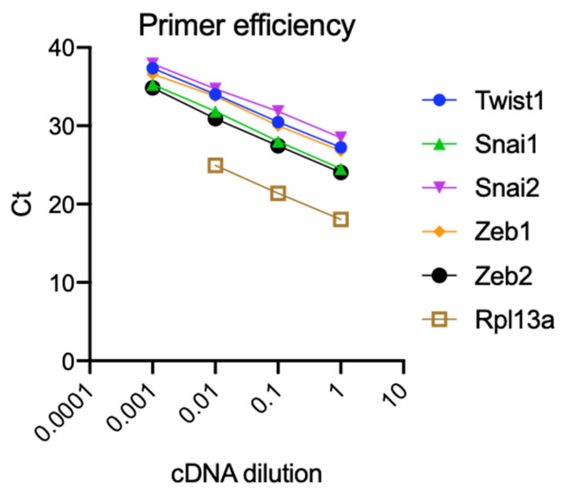

Figure S3. Analysis of primer efficiency performed by plotting the cycle threshold value (Ct) against the serial 1:10 dilution of the cDNA sample.

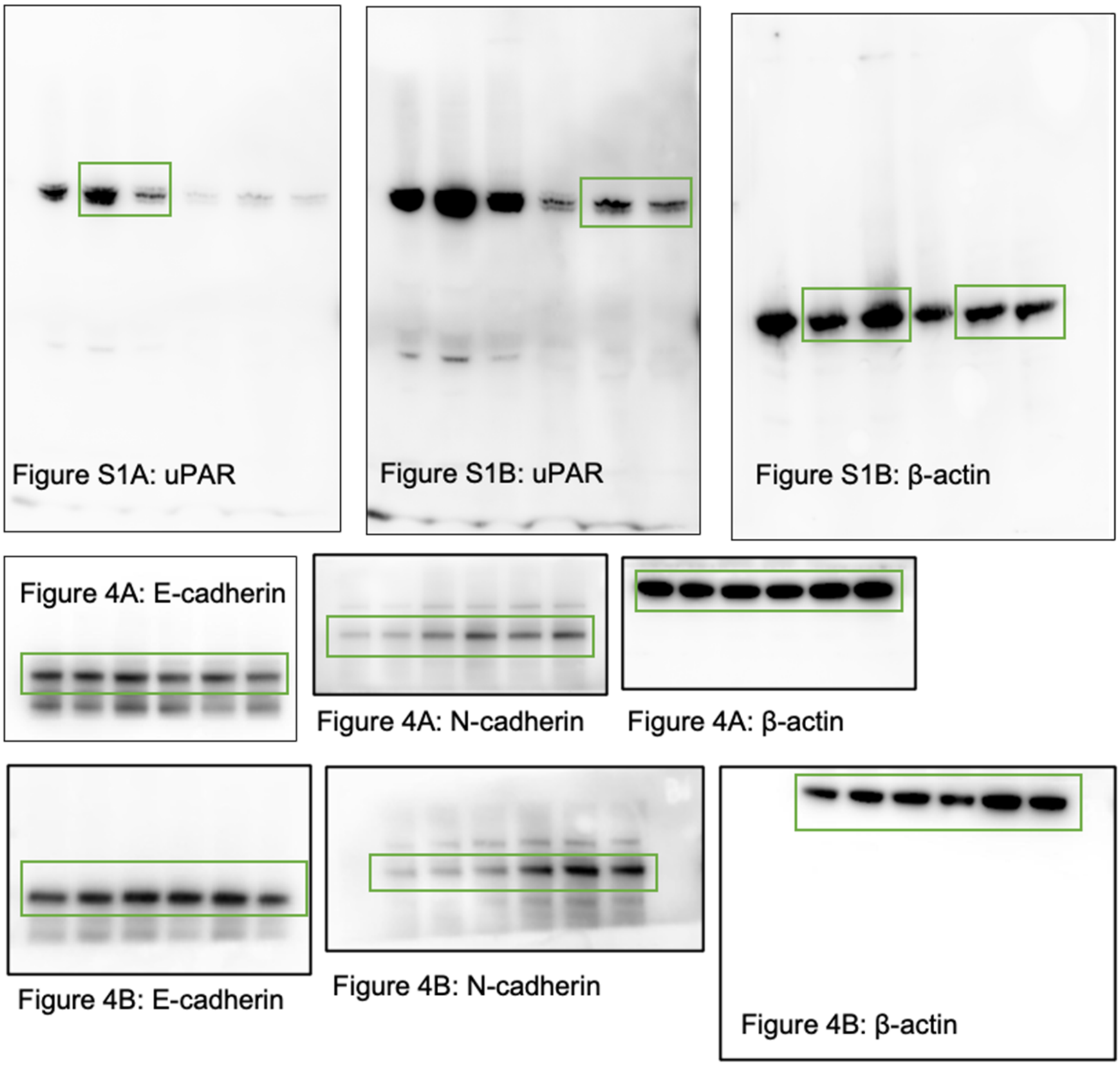

Figure S4. The original uncropped western blot images.

Supplementary tables

Table S1. *PLAU* and *PLAUR* minimal, mean, median, interquartile range, maximal expression levels in control, COVID-19 and pulmonary fibrosis lung cells.

Table S2. shRNA sequences used for cloning.

DNA oligo	Sequence (5' → 3')
PLAUR shRNA F	CCGGGCCGTTACCTCGAATGCATTTCTCGAGAAATGCATTCGAGGTAACGGCTTTTTG
PLAUR shRNA R	AATTCAAAAAGCCGTTACCTCGAATGCATTTCTCGAGAAATGCATTCGAGGTAACGGC
scramble shRNA F	CCGGCCTAAGGTTAAGTCGCCCTCGCTCGAGCGAGGGCGACTTAACCTTAGGTTTTTG
scramble shRNA R	AATTCAAAAACCTAAGGTTAAGTCGCCCTCGCTCGAGCGAGGGCGACTTAACCTTAGG

Table S3. The murine cDNA primers used in the study.

Primer	Sequence (5' → 3')	Primer efficiency	Reference
The murine cDNA primers			
Acta2 F	CGAAACCACCTATAACAGCATCA		[78]
Acta2 R	GCGTTCTGGAGGGGCAAT		
Cdh1 F	CAGGTCTCCTCATGGCTTTGC		[79]
Cdh1 R	CTTCCGAAAAGAAGGCTGTCC		
Cdh2 F	AGCGCAGTCTTACCGAAGG		[79]
Cdh2 R	TCGCTGCTTTCATACTGAACTTT		
Il6 F	GCCCACCAAGAACGATAGTCA		
Il6 R	ACTGGATGGAAGTCTCTT GC		
Plau F	ATGGAAATGGTGACTCTTACCGA		
Plau R	TGGGCATTGTAGGGTTTCTGA		
Rpl13a F	CCCCAGGTAAGCAAACTTTCTG	1.95	
Rpl13a R	CCCCACAAGACCAAGAGAGG		

Snai1 F	CTGCTTCGAGCCATAGAACTAAAG	1.98	
Snai1 R	GAGGGGAACTATTGCATAGTCTGT		
Snai2 F	CTCACCTCGGGAGCATACAGC	2.10	
Snai2 R	TGAAGTGTCAGAGGAAGGCGGG		
Twist1 F	CGGGTCATGGCTAACGTG	1.98	
Twist1 R	CAGCTTGCCATCTTGGAGTC		
Twist2 F	CGCTACAGCAAGAAATCGAGC		
Twist2 R	GCTGAGCTTGTCAGAGGGG		
Zeb1 F	GCTGGCAAGACAACGTGAAAG	2.00	
Zeb1 R	GCCTCAGGATAAATGACGGC		
Zeb2 F	CCACGCAGTGAGCATCGAA	1.90	
Zeb2 R	CAGGTGGCAGGTCATTTTCTT		
